# Abundance, complexation, and trafficking of Wnt/β-catenin signaling elements in response to Wnt3a

**DOI:** 10.1186/1750-2187-2-11

**Published:** 2007-10-25

**Authors:** Noriko Yokoyama, Dezhong Yin, Craig C Malbon

**Affiliations:** 1 Department of Pharmacology, Health Sciences Center, State University of New York at Stony Brook, Stony Brook, NY 11794-8651, USA; 2 Aastrom Biosciences, Inc. Ann Arbor, MI 48105, USA

## Abstract

**Background:**

Wnt3a regulates a canonical signaling pathway in early development that controls the nuclear accumulation of β-catenin and its activation of Lef/Tcf-sensitive transcription of developmentally important genes.

**Results:**

Using totipotent mouse F9 teratocarcinoma cells expressing Frizzled-1 and biochemical analyses, we detail the influence of Wnt3a stimulation on the expression, complexation, and subcellular trafficking of key signaling elements of the canonical pathway, *i.e.*, Dishevelled-2, Axin, glycogen synthase kinase-3β, and β-catenin. Cellular content of β-catenin and Axin, and phospho-glycogen synthase kinase-3β, but not Dishevelled-2, increases in response to Wnt3a. Subcellular localization of Axin in the absence of Wnt3a is symmetric, found evenly distributed among plasma membrane-, cytosol-, and nuclear-enriched fractions. Dishevelled-2, in contrast, is found predominately in the cytosol, whereas β-catenin is localized to the plasma membrane-enriched fraction. Wnt3a stimulates trafficking of Dishevelled-2, Axin, and glycogen synthase kinase-3β initially to the plasma membrane, later to the nucleus. Bioluminescence resonance energy transfer measurements reveal that complexes of Axin with Dishevelled-2, with glycogen synthase kinase-3β, and with β-catenin are demonstrable and they remain relatively stable in response to Wnt3a stimulation, although trafficking has occurred. Mammalian Dishevelled-1 and Dishevelled-2 display similar patterns of trafficking in response to Wnt3a, whereas that of Dishevelled-3 differs from the other two.

**Conclusion:**

This study provides a detailed biochemical analysis of signaling elements key to Wnt3a regulation of the canonical pathway. We quantify, for the first time, the Wnt-dependent regulation of cellular abundance and intracellular trafficking of these signaling molecules. In contrast, we observe little effect of Wnt3a stimulation on the level of protein-protein interactions among these constituents of Axin-based complexes themselves.

## Background

Wnt proteins are secreted, glycosylated and palmitoylated ligands that bind to members of the Frizzled (Fz) family of G protein-coupled receptors and direct aspects of early development [1-9]. Defects in Wnt signaling are implicated in numerous human diseases, *e.g.*, cancer [10] and osteoporosis [11]. Upon binding of Wnt to Frizzled-1 and its co-receptor LRP5/6, a signal is transduced ultimately to the cytoplasmic phosphoprotein, Dishevelled (Dsh/Dvl) [12]. Fly Dsh and the three isoforms of Dvls found in mammals (Dvl1-3) all share three prominent, highly conserved domains: a Dsh homology domain called DIX; a conserved sequence element with homology to the postsynaptic density protein *P*SD-95, *D*isc-large, and *Z*O-1, termed PDZ; and *D*isheveled, *E*gl-10, *P*leckstrin domain, termed DEP [13]. At the level of Dsh/Dvl, signals branch to at least three distinct pathways, namely canonical, non-canonical, and planar cell polarity (PCP) [14].

The canonical Wnt/β-catenin pathway stimulates stabilization and accumulation of cytosolic, and then later of nuclear β-catenin, which binds to Lef/Tcf-sensitive transcription factors and activates genes necessary for early development [15]. In the fly as in the mouse, Wnt3a binds Frizzled-1 (Fz1) and co-receptor LRP5/6, activates Dsh/Dvl which, in turn, suppresses the activity of a key protein kinase, glycogen synthase kinase-3β (GSK3β) [9]. β-catenin, Dishevelleds, and GSK3β appear to interact in a multiprotein Axin based complex which includes the product of the *adenomatous polyposis coli *(APC) tumor suppressor gene [16,17]. Dvl is required for Wnt-dependent inhibition of this complex. Wnt stimulates changes in the abundance as well as the nucleo-cytoplasmic shuttling of β-catenin and perhaps other key signaling elements in the canonical pathway [18,19]. In the absence of biochemical quantification, however, it is not clear whether changes in nucleo-cytoplasmic shuttling observed by confocal microscopy involve 50% or 5% of the cellular complement of these signaling elements, information critical to a full understanding Wnt action at the biochemical level. Developing a quantified measurement of the abundance, localization, and complexation of key elements in response to Wnt thus is an essential goal.

In the current study, we set out to ascertain the answers to three interrelated, unresolved questions: what is the quantified cellular distribution of key elements Axin, Dvl2, GSK3β, and β-catenin in mammalian cells?; how does stimulation by Wnt3a alter their relative abundance and trafficking?; and, are these key protein complexes dynamic and subject to change in response to stimulation by Wnt? Addressing these central questions required use of classical techniques of subcellular fractionation and protein quantification as well as bioluminescence resonance energy transfer (BRET) measurements between protein partners. Our study provides quantification of the relative abundance, trafficking, and complexation of several key signaling elements, exploiting totipotent mouse F9 teratocarcinoma cells expressing Frizzled-1 that form primitive endoderm in response to stimulation with Wnt3a.

## Results

### Wnt3a stimulates changes in cellular abundance of several key signaling molecules in the canonical Wnt/β-catenin pathway

The mouse F9 teratocarcinoma (F9) cells are embryonal and totipotent cells that can be stimulated to form primitive endoderm [6]. Wild-type F9 cells, essentially lacking Rfz1 mRNA, are resistant to the effects of purified Wnt3a and provide an ideal system in which to express Frizzled-1 to study Wnt3a signaling. F9 cells were transfected with an expression vector harboring rat Frizzled-1 (Rfz1) [6]. These Rfz1-expressing F9 cells were adopted as a model system with which to probe biochemical features of the influence of Wnt3a on the abundance, localization, and complexation of key signaling elements of the canonical Wnt/β-catenin pathway.

**Figure 1:**
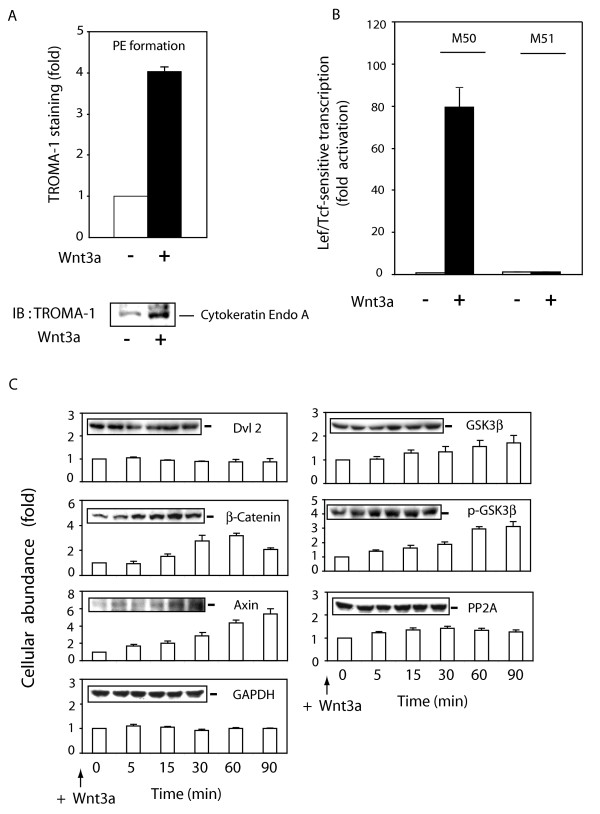
**Wnt3a stimulates changes in cellular content of Wnt/β-catenin pathway signaling components**. *Panel A*, formation of PE was assayed in F9 cells expressing Rfz1 and stimulated without or with purified Wnt3a for 3 days. PE formation was assayed by measuring of expression of PE-marker, cytokeratin endo A, using immunoblotting and staining with TROMA-1 antibody. The results shown are mean values ± S.E. from 3 independent experiments. A representative immunoblot is shown and the mean values of the quantification displayed in the graph. *Panel B*, activation of Lcf/Tcf-sensitive transcription was assayed in F9 cells co-transfected for one-day with Rfz1 and Super8xTOPFlash (M50) or Super8xFOPFlash (M51) and then stimulated without and with purified Wnt3a for 8 hr. The luciferase gene reporter was assayed and is displayed relative to the unstimulated cells (set to 1). The results shown are mean values ± S.E. from 5 independent experiments. *Panel C*, cellular abundance of Axin, Dvl2, GSK3β, p-GSK3β, PP2A C, and β-catenin was assayed in F9 cells expressing Rfz1 and stimulated with Wnt3a for 0 to 90 min. Cells were harvested and lysed in a lysis buffer [50 mM Tris-HCl, pH 7.5, 150 mM NaCl, 5 mM EDTA, 2 mM Na_3_VO_4_, 50 mM NaF, 1 % Triton X-100, 1 mM phenymethysulfonyfluoride (PMSF), 10 μg/ml leupeptin, and 10 μg/ml aprotinin]. Protein expression was established by SDS-PAGE, immunoblotting, and densitometric scans of the immune complexes. A representative blot is shown and the quantification of results is shown as mean values ± S.E. from 4 independent experiments.

Treating the F9 cells with purified Wnt3a stimulates the formation of primitive endoderm (fig. 1A). Primitive endoderm formation was observed within 3–6 days of treatment with Wnt3a, established by immunoblotting of the PE-specific marker, cytokeratin Endo-A. Staining of blots of whole-cell lysates of Wnt3a-stimulated F9 cells with the TROMA-1 monoclonal antibody to Endo-A reveals increased expression of Endo-A. Wnt3a provokes a 4-fold increase in the expression of the PE-marker. Activation of Lef/Tcf-sensitive transcription by β-catenin provides an earlier and robust read-out of the Wnt-stimulated canonical pathway [10]. We analyzed the activity of a Lef/Tcf-sensitive reporter gene, the M50 construct, which harbors multiple β-catenin binding sites in its promoter (fig. 1B). At 8 hr post stimulation with Wnt3a, a robust activation (~80 fold) of the M50 reporter gene was obvious. The activity of the control gene reporter M51, which lacks the β-catenin binding sites [20], was unaffected by treatment with Wnt3a. Activation of Lef/Tcf-sensitive transcription of F9 cells in response to Wnt3a could be detected as early as 4 hr; Wnt5a, in contrast, did not activate Lef/Tcf-sensitive transcription (results not shown).

We intentionally focused our analysis on several key elements of the Wnt canonical pathway, namely Axin, Dvl2, β-catenin, and GSK3β (both native and phospho-forms), whose trafficking has been reported to be regulated by the Wnt canonical pathway [21]. Confocal microscopy reveals changes in apparent localization of these signaling elements, but cannot provide quantitative information. Analysis of trafficking in the absence of detailed cellular quantification of the protein (especially if its abundance is influenced by Wnt stimulation) requires a more detailed analysis of protein abundance as well as subcellular locale. To accomplish this task, we first needed to establish the cellular abundance for each of the signaling elements in whole-cell lysates (fig. 1C). F9 cell lysates were subjected to SDS-PAGE analysis and the resolved proteins to immunoblotting. The blots were stained with antibodies against Axin, β-catenin, Dvl2, GSK3β (phospho- and dephospho-forms), the phosphoprotein phosphatase 2A (*i.e*., the conserved catalytic or "C"-subunit) as well as for GAPDH (as a protein loading control). The F9 cells were either untreated (time = 0) or treated with purified Wnt3a for 5 to 90 min and then subjected to disruption, SDS-PAGE, and quantification of cellular protein (via immunoblotting), setting the abundance of each protein established at zero time as "1" (fig. 1C).

Axin and β-catenin display Wnt3a-induced accumulation over the 90 min stimulation (fig. 1C). At 60–90 min post stimulation by Wnt3a, the abundance of β-catenin is increased 3-fold, the abundance of Axin is increased 4- to 5-fold. The abundance of the phospho-GSK3β also increases, but much more modestly (~50%), in response to Wnt3a stimulation. The abundance of Dvl2, total GSK3β, the protein phosphatase-2A (PP2A), and the loading control GAPDH, in contrast, were essentially unaffected by treating the cells with Wnt3a for periods up to 90 min.

### Biochemical characterization of subcellular fractions

**Figure 2:**
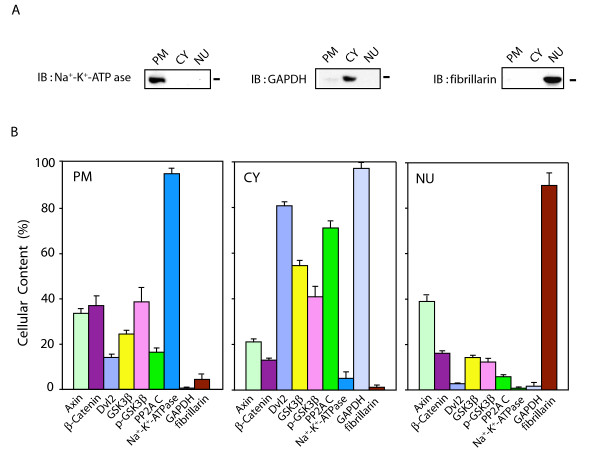
**Cellular distribution of Wnt/β-catenin signaling elements**. Large-scale cultures of mouse F9 cells expressing Rfz1 were harvested and the cells disrupted. Subcellular fractions were prepared from the cell masses and the fractions probed for enrichment in plasma membrane (PM), cytoplasm (CY), and nuclei (NU) as described in detail in the *Methods*. Samples of the whole-cell homogenate were subjected to subcellular fractionation and their complements of cellular proteins to SDS-PAGE. *Panel A*, the distribution of well-known marker proteins for subcellular fractions (*i.e*., Na^+^-K^+^-ATPase as a marker for plasma membrane, GAPDH as a marker for cytoplasm, and fibrillarin as a marker for nuclei) also was analyzed in each fraction to establish purity/enrichment of markers in these fractions. *Panel B*, resolved proteins were analyzed by immunoblotting, stained with one of the following antibodies: anti-Axin, anti-β-catenin, anti-Dvl2, anti-GSK 3β, anti-p-Ser (9)-GSK 3β, and anti-PP2A C-subunit. The relative amounts of the proteins distributed in each subcellular fraction was established densitometrically, based upon their distribution (%), the total protein content of the homogenate and fractions, and quantified analysis of blots from SDS-PAGE. The results are shown as mean values ± S.E. from 8-10 independent experiments.

Results from the analysis of the cellular complement of the signaling elements by immunoblotting clearly demonstrates the need for subcellular fractionation and characterization of the abundance of each of the proteins, in advance of determinations of apparent "protein shuttling" in response to Wnt. Subcellular fractionation of F9 cells was performed by standard protocols and the fractions characterized biochemically for enrichment in marker proteins. Nuclei (NU), plasma membrane (PM), and cytosol (CY, see *Methods*) subcellular fractions were prepared and characterized for their enrichment by immunoblotting and by staining of samples of fractions with antibodies to marker proteins: Na^+^-K^+^-ATPase for plasma membrane; glyceraldehyde-3-phosphate dehydrogenase (GAPDH) for cytoplasm; and the nucleolar-specific protein fibrillarin for nuclei (fig. 2A). The results from the immunoblotting testify to the enriched character of each subcellular fraction prepared from these cells (fig. 2A, B). Each fraction was highly enriched in the appropriate marker for the subcellular source and relatively devoid of marker proteins for the other subcellular fractions.

To complete the biochemical characterization, the distribution of cellular protein among the subcellular fractions prepared from mouse F9 cells was determined (Table 1). Nearly 50% of cellular protein is found in the cytosolic fraction. As commonly observed for subcellular fractionations of other mammalian cells [22], the plasma membrane-enriched fraction displayed ~30% and nuclear-enriched fraction 12–13% of the cellular protein. The sum of mitochondria-enriched fraction and of high-speed, supernatant "microsomal" fraction, together representing ~10% of the cellular protein and essentially devoid of markers for plasma membrane, cytoplasm, or nucleus, were not further probed in these studies. The amount of the marker proteins and signaling elements established empirically in the plasma membrane + cytoplasm + mitochondria + microsomes + nuclear-enriched fractions was set to 100% for all derivative calculations of signaling element distribution (Table 1).

**Table 1:** Protein distribution in subcellular fractions prepared from mouse F9 cells. Pre-confluent F9 cells were harvested from P150 cultures, homogenized, and subcellular fractions prepared for analysis of relative protein content, as outlined in the *Methods*. The sum of these 4 subcellular fractions was set as "100 %". The data displayed are the mean values ± S.E.M from separate preparations (*n *= 20).

Fractions	Protein distribution %	Protein (mg)
Nuclei	12.25 ± 0.450	01.81
Mitochondria+Microsomes	10.64 ± 0.347	01.57
Plasma Membrane	30.06 ± 1.078	04.45
Cytoplasm	47.05 ± 0.332	06.96

Total	100	14.79

### Cellular distribution of Wnt/β-catenin pathway signaling elements

Determination of "specific activity" (relative content of target molecule/mg protein of sample, fig. 2), cellular protein content (Table 1), and fraction volumes (*Methods*), enabled us to establish the level of relative abundance and subcellular localization of Axin, β-catenin, Dvl2, GSK3β and PP2A in mouse F9 cells (fig. 2B). To optimize the data acquisition, densitometric analyses were performed on a UMax 1000 absorbance scanner equipped with SilverFastAi software and quantified using Aida software. Distributions of proteins were calculated based on the protein concentration, fraction volume, and quantification of blots under conditions in which ~95 % of marker proteins for plasma membrane, cytosol, and nucleus are each found in the appropriate subcellular fraction. The data are the results of more than nine large-scale preparations of F9 cells, each assayed on independent occasions. This is the first such report to quantify the abundance and subcellular distribution of these key elements of the Wnt canonical pathway (fig. 2B).

Axin, in combination with the *adenomatous polyposis coli *(APC) gene product, has been shown act as a scaffold that chaperones β-catenin, shuttling to and from the nucleus [23]. Axin expression in some cells and the absence of Wnt appears to be lower than that of the other proteins in the multiprotein complex responsible for the degradation of β-catenin [24], but was detectable in each of the subcellular (plasma membrane-, cytosol-, and nuclear-enriched) fractions prepared from the mouse F9 cells. We established the presence of significant amounts of Axin in all three subcellular fractions. Approximately 35% of Axin was associated with the plasma membrane; > 40% in nuclear fraction; the remainder in the cytosol accounting for < 25%.

β-catenin, in contrast, is found most abundant in the plasma membrane-enriched fraction, likely in association with the cell membrane-associated cadherins [25] and Axin (fig. 2B). β-catenin was found at significant levels in the cytosol, again perhaps in combination with its chaperone Axin. Nuclear fractions displayed the least content (about 15%) of the cellular β-catenin, in the absence of Wnt3a.

In unstimulated cells, Dishevelled-2 is found predominantly (> 80%) in the cytosol-enriched subcellular fraction (fig. 2B). Approximately 10–15% of this phosphoprotein is found associated with the plasma membrane (fig. 2B). In the fly, Dsh (homologue of mammalian Dvl) has been shown to harbor both a nuclear localization sequence (NLS) at carboxy-terminal to the PDZ domain as well as a nuclear export signal (NES) at the carboxy-terminal reach of the DEP domain [18]. Dishevelled-2, which does not display canonical mammalian sequences for either NLS or NES, was found to be the least abundant in the nucleus, displaying less than 3% of the cellular complement in cells absent stimulation by Wnt.

The serine/threonine-specific protein kinase glycogen synthase kinase-3β (GSK3β) plays a pivotal role in the Wnt canonical pathway [26], catalyzing the phosphorylation of each of the key signaling elements under study herein. The amount of GSK3β was found to be greatest in the cytosol (> 50%, fig. 2B), although it was readily detected in the plasma membrane-enriched and nuclear subcellular fractions. High levels of GSK3β activity have been reported in the nuclei of primary cortical neurons of mouse brain [27], although the relative abundance of this kinase in subcellular fractions of embryonic cells has not been reported. The cellular distribution of active GSK3β and its phosphorylated, inactive form (p-GSK3β) among the three major subcellular fractions was found to be essentially the same in the unstimulated F9 cells.

PP2A, a serine/threonine phosphatase, implicated in the Wnt canonical pathway [28], was found localized predominantly (> 70 %) in the cytosolic fraction. A significant amount of this phosphoprotein phosphatase, however, was found also in the plasma membrane-enriched fraction. Only a small amount of PP2A (~5%) was found in the nuclear fraction prepared from unstimulated cells. Of note, the cellular distributions of PP2A and Dvl2 in unstimulated cells appear to be quite similar, suggesting the possibility of some close association between these two key molecules. Establishing a detailed quantification of abundance and subcellular distribution by biochemical means in the unstimulated F9 cells enabled us to proceed to an analysis of the trafficking of these key signaling molecules in response to stimulation of the canonical pathway by treatment with Wnt3a.

### Wnt3a stimulates a rapid trafficking of Wnt canonical signaling elements

**Figure 3:**
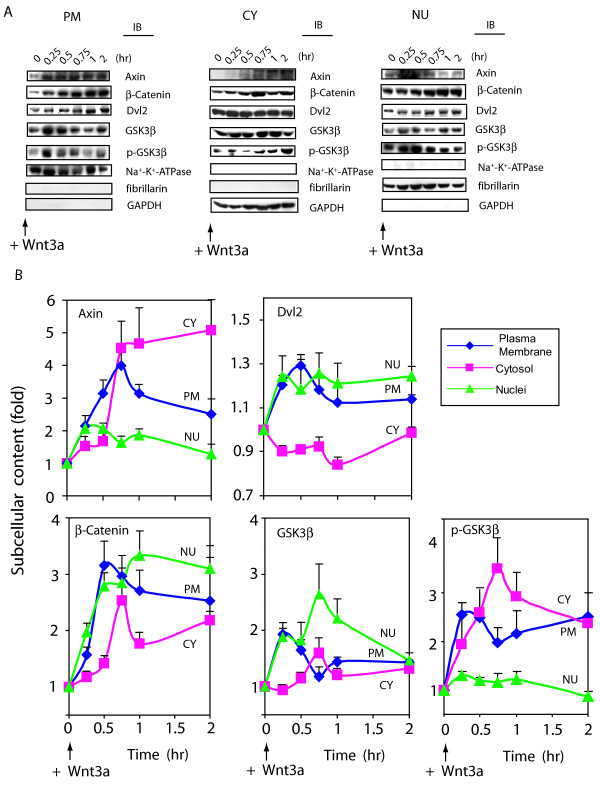
**Wnt3a stimulates a rapid shuttling of Wnt/β-catenin signaling elements**. *Panel A*, quantified immunoblot analysis of subcellular fractions obtained from control cells (time = 0) and cells stimulated with purified Wnt3a. F9 cells expressing Rfz1 receptor were stimulated with Wnt3a for the indicated times. Cell cultures were collected, disrupted, and fractionated to the plasma membrane, cytoplasm and nuclei fractions, as described in *Methods*. Each fraction (100 μg) was subjected to SDS-PAGE and analyzed by immunoblotting with specific antibodies targeting the signaling molecules indicated. The *three panels *displayed at the bottom of the immunoblot set show blots stained with antibodies to well known subcellular marker proteins: Na^+^-K^+^-ATPase (plasma membrane), GAPDH (cytoplasm) and fibrillarin (nuclei), respectively. The result shown is representative of 10 independent experiments. *Panel B*, summary of the quantified trafficking of signal elements in response to Wnt3a. Bands were quantified by densitometry as described in *Methods *and values are displayed as fold of zero time point. The content of key signaling molecules in the plasma membrane- (PM, blue line), cytoplasmic- (CY, pink line) and nuclear- (NU, green line) enriched subcellular fractions are displayed. The results are shown as mean values ± S.E. from 6–10 independent experiments.

Our overarching goal was to quantify the trafficking of key signaling molecules of the Wnt/β-catenin pathway in response to Wnt3a stimulation. Within 90 min of stimulation with Wnt3a, F9 cells display a substantial increase in the abundance of Axin, of β-catenin, and to a lesser extent of the protein kinase GSK3β (fig. 1C). We incorporated the changes in cellular abundance, referencing the subcellular localization established for each molecule in the absence of Wnt (*i.e.*, the starting time, t = 0) and quantified the cellular distribution of the Axin, β-catenin, Dvl2 and GSK3β in F9 cells treated with Wnt3a (fig. 3). The cells were stimulated with purified Wnt3a for periods up to two hours. Immunoblots of subcellular fractions prepared from large-scale growths of F9 cells treated with Wnt3a at various times from a representative experiment are displayed for each signaling molecule (fig. 3A). The relative changes in distribution of the signaling molecules among the subcellular fractions obtained from 6–10 individual, separate experiments were compiled, analyzed statistically, and displayed (fig. 3B).

Stimulation of Wnt3a initially provokes a major recruitment of Axin, β-catenin, Dvl2, and GSK3β to the plasma membrane (fig. 3A, B). For Dvl2 and GSK3β, the transit to the plasma membrane-enriched fraction in response to Wnt3a is very rapid, being greatest at the earliest time point measured, 15 min post stimulation. For Axin, a scaffold for the multiprotein complex that targets β-catenin for degradation, as well as for β-catenin itself, Wnt3a stimulates a rapid increase in plasma membrane-association, easily detected within 15 min and reaching peak values by 45 min post stimulation. The recruitment of Axin to the plasma membrane- associated LRP5/6 co-receptor of Fz1 has been shown to be mediated by a rapid phosphorylation of LRP5/6 by CK1γ as well as by GSK3β [29,30]. Dvl2 shuttles to the plasma membrane-enriched fraction in response to stimulation by Wnt3a, was readily observed at 15 min, attaining peak values within 30 min.

This biochemical, quantified data extend earlier results obtained largely from immunofluoresence techniques [31,32]. The similarity in the relative increases and temporal coincidence of the changes noted suggest that Axin, Dvl2, and GSK3β are trafficked *en masse *to the plasma membrane-enriched fraction in response to Wnt3a stimulation. Unlike the response observed for Dvl2 and GSK3β, whose abundance does not change with Wnt stimulation, the amount of Axin and β-catenin associated with the plasma membrane continues to increase beyond 15 min, for nearly 60 min. Increased stabilization in response to inhibition of the degradation complex and/or increased synthesis of these two molecules adds substantially to the pool of Axin and β-catenin at the plasma membrane (fig. 3B). A sustained accumulation of Axin occurs in the cytosol in response to stimulation by Wnt3a; this accumulation then accounts for much of the cellular content of this scaffold. Trafficking of Axin to the nuclear-enriched fraction increases to a lesser extent (two-fold) in response to Wnt3a, transiently returning to normal within 120 min. Phosphorylation of GSK3β on Ser9 (detected by immunoblotting with a phospho-specific antibody for p-GSK3β) was coincident with the movement of GSK3β to the plasma membrane, providing compelling although indirect evidence that inactivation of GSK3β in response to Wnt3a may occur with trafficking of this kinase to the plasma membrane. Although Ser-9 phosphorylation has been difficult to detect, increased phosphorylation of peptides corresponding to that harboring the Ser-9 residue has been reported in response to Wnt [33,34]. In addition, the serine-9 to alanine mutant of GSK3β was found to be 2-fold better than the wild-type at blocking hDvl2-induced activation of the Lef/Tcf-sensitive transcriptional response [33]. In F9 cells, enhanced phosphorylation of GSK3β Ser9 is observed in both the cytosol-enriched and the plasma membrane-enriched fractions at earliest time point measured, 15 min post stimulation by Wnt3a (fig. 3).

In the cytosol, Wnt3a stimulates a sustained 5-fold increase in Axin as well as a more modest increase in β-catenin (fig. 3B). The increase in cytosolic Axin in response to Wnt3a follows temporally the marked and transient increase in plasma membrane-associated Axin. For β-catenin, cytosolic levels increase in response to Wnt3a, lagging temporally behind sustained major increases in β-catenin accumulation at the plasma membrane and in the nucleus. Wnt3a stimulates a modest, transient increase in cytosolic GSK3β, while cytosolic Dvl2 levels essentially decline in response to the increased trafficking of Dvl2 to the plasma membrane-enriched and nuclear-enriched subcellular compartments.

With regard to shuttling to the nucleus, Wnt3a stimulates initial 2-fold and 3-fold increases in Axin and β-catenin, respectively (fig. 3B). The increase in nuclear Axin in response to Wnt3a stimulation was sustained for one hour, whereas the increase in nuclear β-catenin persisted to 2 hr, the last time point analyzed. Axin is reported to chaperone β-catenin, facilitating both import to and export from the nucleus [35-37]. Study of protein-protein interactions in live cells using analysis such as energy transfer may reflect the strength (*i.e.*, distance) or temporal character of the Axin-β-catenin interaction and might be useful in this regard (see below). Thus some portion of the β-catenin may only be interacting with Axin as Axin shuttles β-catenin to the nucleus, acting as a "chaperone". GSK3β, like β-catenin, displays nuclear localization, although a transient accumulation rather than one sustained over 120 min in response to Wnt3a. Wnt3a provokes GSK3β to associate transiently in the plasma membrane, a recruitment that peaks within 15 min of stimulation (Fig. 3A and 3B). Wnt3a also stimulates trafficking of GSK3β to the nucleus. Finally, Dvl2 shows a modest, but sustained trafficking to the nucleus in response to stimulation with Wnt3a. Wnt3a has been reported to stimulate the shuttling of some Dsh to the nucleus in *Xenopus *embryonic explants [18]. We demonstrate for the first time in mammalian cells that cytoplasmic to nuclear shuttling of a major portion (~30%) of Dvl2 can be observed in the mouse F9 embryonal cells stimulated with Wnt3a (figs. 2, 3).

### BRET analysis of protein-protein interactions in situ among key signaling molecules in the Wnt/β-catenin pathway

**Figure 4:**
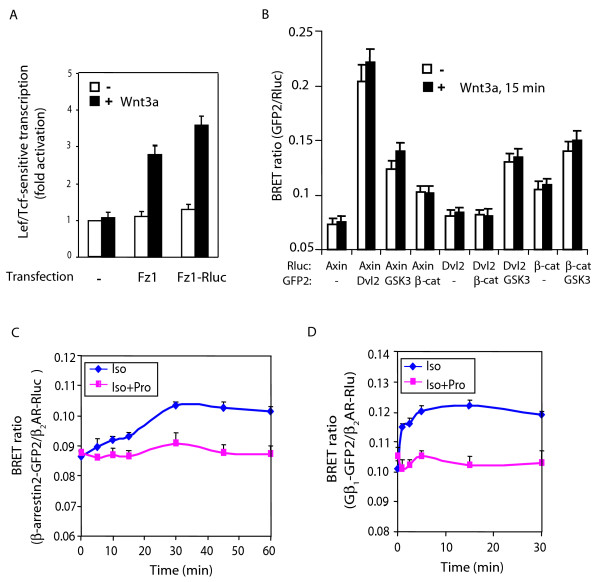
**BRET^2 ^analysis of protein-protein interactions in the Wnt/β-catenin signaling pathway**. *Panel A*, Lef/Tcf-sensitive gene activation in HEK 293 cells in response to Wnt3a. HEK 293 cells were co-transfected with either Rfz1 and M50 or Rfz1-Rluc and M50. HEK 293 cells were stimulated with Wnt3a (20 ng/ml) for 8 hr. Lef/Tcf-sensitive transcription activity was determined. *Panel B*, HEK 293 cells stably expressing Rfz1 were transiently co-transfected with Axin-Rluc and/or Dvl2-GFP2, Dvl2-Rluc, GSK3-GFP2, β-cat-GFP2, β-cat-Rluc as indicated for 48 h and then treated with Wnt3a (20 ng/ml) for 15 min. BRET ratios were measured by addition of DeepBlue C (5 μM) in cells co-expressing Rluc and GFP2 fusion proteins. Results are expressed as the mean ± S.E. of at least three independent experiments. *Panel C*,the β-adrenergic agonist isoproterenol (Iso) stimulates the interaction between GPCR and β-arrestin2. HEK293 cells were transiently transfected with β_2_AR-Rluc and β-arrestin2-GFP2 for 48 h, and then treated with Iso (10 μM) or Iso plus the β-adrenergic antagonist propranolol (Pro, 10 μM) for 0 to 60 min. *Panel D*, Iso stimulates the interaction between GPCR and the heterotrimeric G protein subunit Gβ_1_. HEK293 cells were transiently transfected with β_2_AR-Rluc, Gβ_1_-GFP_2_, Gα_s_, and Gγ_2 _for 48 h, and then treated with agonist Iso (10 μM) or Iso plus antagonist Pro (10 μM) for 0 to 30 min. BRET ratios (GFP2/Rluc activity) were measured by addition of DeepBlue C (5 μM) in cells coexpressing Rluc and GFP2 fusion proteins. Results are expressed as the mean ± S.E. of three independent experiments. Note that isoproterenol stimulates GPCR interaction with G-protein first (peaks within 5 min of agonist) and later leads to association of GPCR with β-arrestin2 (peaks at 30 min). Both sets of protein-protein interactions are blocked by simultaneous addition of propranolol with agonist.

Our data suggest a close, perhaps "coupled" interaction of Axin with Dvl2 and β-catenin during activation of the canonical pathway by Wnt3a in mouse F9 cells (figs. 2, 3). As early as 15 min following stimulation of the cells with Wnt3a, we observe strikingly similar patterns of trafficking of all three signaling elements to the plasma membrane- and to the nuclear-enriched subcellular fractions (fig. 3B). Not so for the results obtained by analysis of the content of these molecules in the cytosol-enriched subcellular fractions, likely reflecting the differential effects of Wnt3a on the cellular abundance, rather than shuttling for both Axin and β-catenin. To probe this question more directly and *in situ *in live cells, we made use of bioluminescence response energy transfer (BRET) in which biomolecular interactions can be probed in live cells [38]. The BRET partners of R-luciferase-fusion proteins and eGFP2-fusion proteins of Axin, Dvl2, and β-catenin were engineered and expressed in various combinations in cells co-expressing Rfz1. Despite repeated efforts, we were not able to express the BRET fusion proteins in F9 cells at the levels that are necessary to provide unambiguous results of protein-protein interaction (results not shown). As an alternative, we employed HEK 293 cells that display a robust synthetic and chaperone capacity for exogenously introduced expression vectors [39]. Expression of the necessary fusion protein partners was successful in the HEK 293 cells, permitting BRET^2 ^analysis of protein-protein interactions. BRET^2 ^assay is performed with improved fusion moieties (*e.g.*, GFP2) in combination with a more stable substrate for the *Renilla *luciferase (Rluc) [38,40]. Wnt3a stimulation of HEK 293 cells expressing Rfz1 resulted in robust activation of Lef/Tcf-sensitive luciferase gene reporter (fig. 4A), similar to that observed for F9 cells (fig. 1B). We tested the function of the fusion proteins, exemplified by the analysis of Rfz1-Rluc, which was shown to be fully functional (fig. 4A). The BRET ratio was measured in live cells at 15 min following Wnt3a stimulation (fig. 4B).

We detected a strong BRET^2 ^signal from the Axin/Dvl2 partners (fig. 4B). The individual partners alone (fig. 4B) or in combination with non-fused version of eGFP2 or R-luciferase (controls such as Rluc/GFP2, Axin-Rluc/GFP2, Dvl2-Rluc/GFP2, β-catenin-Rluc/GFP2, Rluc/Dvl2-GFP2, Rluc/GSK3β-GFP2, Rluc/β-catenin *etc.*, result not shown) provide no significant BRET^2 ^signal under these same conditions. Protein-protein interactions of Axin with Dvl2, Axin with GSK3β, as well as Axin with β-catenin, all generated strong BRET^2 ^signals in the unstimulated HEK 293 cells. These BRET signals reflect the intensity (*i.e.*, spatial proximity) of the proteins interacting. Likewise, protein-protein interactions probed for Dvl2 with GSK3β as well as GSK3β with β-catenin generated substantial BRET^2 ^signals in unstimulated cells, indicative of *bona fide *protein-protein interaction between these partners.

Notable, in the cells stimulated with Wnt3a for 15 min, a time point when Wnt-induced shuttling of these key signaling elements is already well underway (fig. 3), no significant change is observed in the magnitude of the BRET^2 ^signals for any of these protein-protein interactions (fig. 4B). Dissociation of partners or a significant decrease in the level of protein-protein interactions in response to Wnt3a would have been predicted to provoke a sharp reduction or total loss of BRET^2 ^signal. These novel data suggest that the interactions of each protein-protein partner (or interactions within a multiprotein complex that include these partners) remain largely intact during trafficking of these key signaling molecules to subcellular fractions is response to Wnt3a (fig. 4). Either dissociation of partners or enhanced association of these protein-protein partners would have been readily detected by BRET analysis. We tested our BRET responses using two positive controls: the agonist-stimulated association of the β_2_-adrenergic receptor with either the adaptor protein β-arrestin2 or the G-protein β1 subunit. We assayed BRET signals for the interaction between the β_2_-adrenergic receptor-GFP2 and β-arrestin2-Rluc in response to addition of a β-adrenergic agonist in these same cells and observed a dynamic changes in BRET signal in response to isoproterenol (Iso, fig. 4C), an interaction that could be blocked by simultaneous treatment with agonist and the β-adrenergic antagonist propranolol (Pro). The lack of change in the BRET^2 ^signal observed in response to Wnt3a is thus striking (fig. 4B). Similarly, using β_2_-adrenergic receptor-Rluc and a trimeric Gα_s _that includes Gβ_1_-GFP2, we readily detected increased BRET signal in response to agonist, an interaction that could be blocked by the β-adrenergic antagonist propranolol (fig, 4D). Note that the observed kinetics of these two distinct protein-protein interactions also confirm the literature, the agonist treatment rapidly leads to GPCR-G protein association (within 5 min) and then later to interaction with β-arrestin2 (peaking at 30 min). Thus, using the same cells and conditions we demonstrate sensitive, real time changes of BRET signals from other GPCR signaling cascades, supporting the value of such determinations for the Wnt/β-catenin signaling cascade. At 15 min post stimulation by Wnt3a, trafficking of Axin, Dvl2, and GSK3β is obvious, whereas at this time the effects of Wnt3a on cellular abundance of the signaling elements are not significantly expressed. Thus, it seems likely that Axin, Dvl2, and GSK3β operate as a relatively stable multi-protein complex and that this complex shuttles to the plasma membrane and to the nucleus in response to Wnt3a.

### Differential localization and trafficking of Dvl1, Dvl2, and Dvl3 in response to Wnt3a

**Figure 5:**
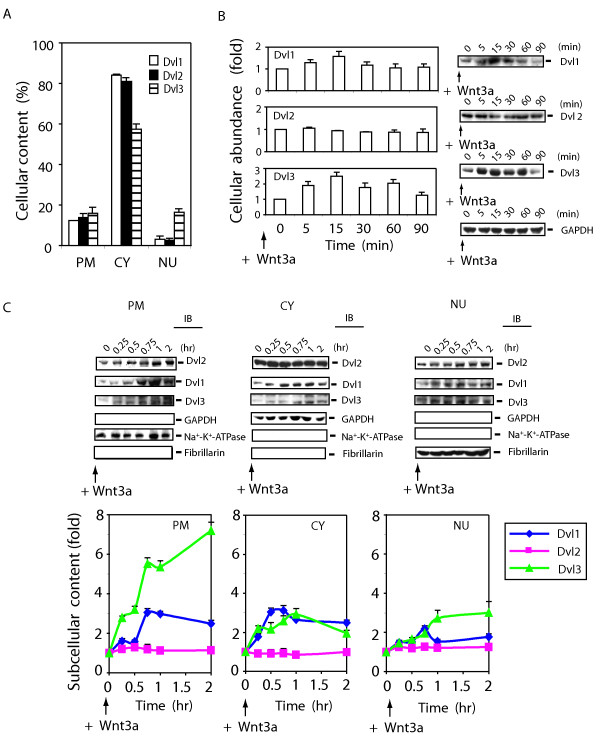
**Differential effects of Wnt3a stimulation on Dvl1, Dvl2, and Dvl3**. *Panel A*, the comparison of the cellular abundance of mammalian Dvl isoforms (Dvl1, Dvl2, and Dvl3) in subcellular fractions. F9 cells expressing Rfz1 were used to prepare subcellular fractions. Plasma membrane-enriched (PM), cytosol-enriched (CY), and nuclear-enriched (NU) fractions were prepared as described in detail in *Methods *and are displayed. The results of multiple densitometric scans of immunoblots were quantified. The results are displayed based upon the distribution (%), the total protein content of the whole-cell homogenate and fractions, and quantified analysis of blots from SDS-PAGE for each molecule and various controls for sample loading and blotting. The results are shown as mean values ± S.E. from 6–8 independent experiments. *Panel B*, the comparison of the cellular abundance of Dvls in response to Wnt3a. F9 cells expressing Rfz1 were stimulated with purified Wnt3a for indicated time and then disrupted in a lysis buffer [50 mM Tris-HCl, pH 7.5, 150 mM NaCl, 5 mM EDTA, 2 mM Na_3_VO_4_, 50 mM NaF, 1 % Triton X-100, 1 mM phenymethysulfonyfluoride (PMSF), 10 μg/ml leupeptin, and 10 μg/ml aprotinin]. Abundance of each Dvl isoform was determined by staining of blots with specific antibodies against each Dvl. Fractions were also stained with anti-GAPDH antibody to establish loading equivalence. Values are displayed as ''fold'' of zero-time point (set to 1). A representative blot is shown and the quantification of results is shown as mean values ± S.E. from 6 independent experiments. *Panel C*, F9 cells expressing Rfz1 receptor were stimulated without or with Wnt3a. Cells were harvested at each time point indicated, cell lysates were fractionated into plasma membrane (PM), cytoplasm (CY) and nuclei (NU) fractions. Quantified immunoblotting was performed as described in *Methods*. Each fraction (100 μg) was subjected to SDS-PAGE and the resolved proteins analyzed by immunoblotting with anti-Dvl1-, anti-Dvl2- and anti-Dvl3-specific antibodies. The *three panels *displayed at the bottom of the immunoblot set show blots stained with antibodies to well known subcellular marker proteins: Na^+^-K^+^-ATPase (plasma membrane), GAPDH (cytoplasm) and fibrillarin (nuclei), respectively. Representative blots are shown (*top panel*). Summary of quantified analysis of blots from SDS-PAGE are shown (*bottom panel*). The results are shown as mean values ± S.E. from 4–6 independent experiments. Dvl1 (blue line), Dvl2 (pink line) and Dvl3 (green line) are displayed.

Unlike the fly which expresses a single Dishevelled (*i.e.*, Dsh), mammalian cells can express three highly homologous isoforms of Dishevelled, termed Dvl1, Dvl2 and Dvl3 [41]. Dvl2 is the most abundant Dvl in mouse F9 cells (results not shown). We explored the cellular distribution of each mammalian Dvl expressed in F9 cells (fig. 5). All three Dvls were expressed in mouse F9 cells, as determined by immunoblotting with isoform-specific antibodies (fig 5A). The cellular content of Dvl1 and Dvl2 among the three subcellular fractions was nearly identical. The bulk of Dvl1 and of Dvl2 (> 80%) is found in the cytosol-enriched fraction, the least amount of each of these isoforms (> 5%) is observed in the nuclear-enriched fractions. Notable differences for the subcellular content and distribution of Dvl3 as compare to either Dvl1 or to Dvl2 were observed. Dvl3 content in the cytosol-enriched fractions is markedly reduced (~30%) in comparison to the content of either Dvl1 or Dvl2. More importantly, the content of Dvl3 in the nuclear-enriched fraction of unstimulated F9 cells is several folds greater than that for either Dvl1 or Dvl2 (fig 5A).

Next we analyzed the abundance of each Dvl in cells stimulated by Wnt3a (fig. 5B). As noted above (fig 1C), the abundance of Dvl2 is unaffected by Wnt 3a stimulation. The abundance of Dvl3 and Dvl1 following stimulation by Wnt3a, in contrast to that of Dvl2, were increased. The abundance of Dvl3 increases 2-fold within 5 min post-stimulation of Wnt3a and reaches to ~2.5 fold within 15 min.

Dvl2, as shown above (fig. 3), shows a Wnt3a-stimulated trafficking to plasma membrane- and nuclear-enriched fractions at the expense of the cytosolic complement of this phosphoprotein. Rapid trafficking of all three Dvls to the plasma membrane-enriched subcellular fraction was observed in response to stimulation by Wnt3a (fig. 5C). The trafficking of Dvl1 and Dvl3 to the plasma membrane and nuclear fractions in response to stimulation by Wnt3a, unlike that of Dvl2, does not appear to occur at the expense of cytosolic complement. Most remarkable is the magnitude of the relative changes of Dvls for Wnt3a-stimulated trafficking to the plasma membrane. Wnt3a stimulates a 7-fold increase in Dvl3 trafficking to the plasma membrane-enriched fraction, a process that continues progressively over the 120 min stimulation by Wnt3a. The Wnt3a-stimulated increase in the abundance of Dvl3 enables this sharp increase in accumulation at the plasma membrane. The trafficking of Dvl1 and Dvl2 in response to Wnt3a stimulation, in contrast, reaches a plateau (3-fold and 1.3-fold, respectively) within 60 min, thereafter declining over the next 60 min (fig. 5C).

## Discussion

The Wnt canonical signaling pathway plays an essential role in early development [2]. The input is Wnt3a which binds to Frizzled-1, a member of the superfamily of the heptihelical G protein-linked receptors that are embedded in the plasma membrane [9,42]. Functional binding of Wnt to Frizzled-1 requires the co-receptor LRP5/6, also a plasma membrane-embedded protein [43]. Thus, signaling from Wnt to the level of activation of gene transcription in the nucleus requires additional protein(s) that can propagate the signal physically from the inner face of the cell membrane to the nucleus. Members of the Dsh/Dvl family interact with the Frizzled/LRP6/Go proteins at the membrane [44] and in combination with a multiprotein complex are responsible for the inhibition of phosphorylation of β-catenin, which targets β-catenin for degradation [9]. Dishevelled has been shown to be downstream of Frizzled/LRP6/Go and upstream of GSK3β signaling of both the canonical and PCP pathways in the fly, through epistasis experiments [8,45]. It is considered that at Dsh/Dvl level, Wnt signals branch to canonical, non-canonical and PCP pathways [14].

Inhibition of GSK3β-catalyzed phosphorylation of β-catenin requires the participation of members of the dynamic multiprotein complex, including APC, Axin, and most prominently the unique phosphoprotein Dsh/Dvl [16,17]. APC, β-catenin and Axin have been shown capable of cycling between the cytoplasm and the nucleus [35-37], although the extent to which these changes involve the full cellular complement of the signaling molecules has remained enigmatic. Elegant earlier studies were based largely upon fluorescence and immunoblotting techniques which indicate changes in locale, [31,32,46], but fail to provide quantification and unambiguous temporal description of shuttling to biochemically-defined compartments of the cell. Phosphorylation plays a key role in both changes in protein stability and localization of these signaling elements [16,47]. In the current work we apply well-validated biochemical techniques to ascertain changes both the cellular abundance and distribution of Axin, Dvls, β-catenin, as well as GSK3β in response to Wnt3a. Our quest was to establish how much of the cellular content of each signaling molecule is being shuttled and to which subcellular fraction in response to activation of the canonical Wnt/β-catenin/Lef-Tcf-dependent transcriptional pathway.

We succeed in establishing the distribution of key signaling elements, defining both symmetric (for Axin, found in all three major subcellular fractions) as well as markedly asymmetric (for Dvl2, mostly found in the cytosol, and β-catenin found most abundant in the plasma membrane) distributions among plasma membrane-, cytosol-, and nuclear-enriched subcellular fractions. Following Wnt3a stimulation, the abundance of Axin, β-catenin and Dvl3 markedly increases, while the abundance of Dvl2 and GSK3β was largely unaffected. The amplitude and temporal character of the shuttling of Axin, Dvl2 and GSK3β to various well-defined subcellular fractions suggest that these proteins may form a relatively stable complex (es) that shuttles ultimately to the nucleus, where both Axin and β-catenin accumulate. Recent structural insights into Dvl and Axin suggest a role for dynamic polymerization of Dvl via its DIX domain [48]. Our data suggests that Dvl2 and Axin form a complex that is stable in the face of Wnt3a stimulation. Although speculation, dynamic polymerization may be of stable complex that contain minimally Dvl and Axin. Axin negatively regulates Wnt canonical signaling, acting as a scaffold to which GSK3β, β-catenin, APC, Dvl, and PP2A can be recruited.

Previous results suggested that Wnt stimulation can promote Axin degradation in mammalian cell culture [49-51] and fly embryos [52]. We show herein a sharp increase of abundance of Axin in response to stimulation by Wnt3a. These earlier studies analyzed the abundance of Axin only after 2–6 hr post stimulation by Wnt. Analysis of Axin changes over short time courses with respect to Wnt stimulation has not been reported, until now. Furthermore, our studies were largely performed on the native signaling molecules operating at wild-type levels of expression, rather than with overexpressed, tagged fusion proteins whose expression levels may well disrupt normal stoichiometries and signaling.

The fly Dsh, displaying both NLS and NES, has been shown to shuttle to the nucleus in response to activation of the Wnt canonical pathway [18]. By high-resolution confocal microscopy and photobleaching techniques, others have shown that Dvl2 (and Axin) forms dynamic "puncta" assemblies absent of cytoplasmic vesicles and equilibrating with cytosolic pools [46]. Nuclear localization of Dsh is required for its function in the canonical Wnt/β-catenin signaling pathway in the fly [18,19], so detailed analysis of the abundance and localization of mammalian Dvls was an essential effort. Biochemical analysis of Dvls distribution in cells stimulated with Wnt3a enabled us to quantify temporally the accumulation of Dvl1, Dvl2, and Dvl3 in the plasma membrane-enriched and nuclear-enriched subcellular fractions. Our results show that the bulk (> 65%) of the Dvl2 remains in the cytosol following stimulation with Wnt3a, with about 20% transiting to the plasma membrane and > 10% of Dvl2 accumulating over time in the nucleus in response to Wnt3a. In contrast, the localization, changes in abundance and trafficking of Dvl1, Dvl2 and Dvl3 in response to Wnt3a are distinct.

Dvl1 and Dvl3 display increased cellular accumulation in response to Wnt3a, Dvl2 does not. Dvl1 and Dvl3 display Wnt3a-stimulated changes in trafficking to the plasma membrane (2.5- and 7.0-fold, respectively), whereas, for Dvl2, the changes are relatively small. Further analysis is essential to elucidate how changes in the cellular abundance of Dvl1 and Dvl3 impact Wnt/β-catenin signaling. Our biochemical data demonstrate Wnt-stimulated trafficking of Dvl isoforms to the nucleus in mammalian cells, similar to nuclear localization of Dsh observed in the fly [18]. Nuclear accumulation of Dvl2 was minimal, whereas nuclear accumulation of Dvl1 was transient, and that of Dvl3 was prominent (~3-fold) in response to stimulation with Wnt3a. Unique differences in cellular distribution of the three Dvls as well as their nuclear localization in response to Wnt3a suggest the possible existence of isoform-specific roles of the highly homologous mammalian Dvl1, Dvl2, and Dvl3 in Wnt/β-catenin signaling pathway.

Analysis of the bimolecular protein-protein interactions in live cells using BRET technology demonstrates strong protein-protein interactions between Axin and Dvl2, between Axin and GSK3β, and between Dvl2 and GSK3β. The cellular localization/trafficking of the partners in these multiprotein complexes is shown biochemically to be regulated by stimulation with Wnt3a. The BRET^2^-derived protein-protein signals from these partner interactions, however, remained essentially unchanged in response to stimulation of the cells with Wnt3a, although Wnt3a-stimulated trafficking among subcellular fractions was obvious.

## Conclusion

This study provides a detailed biochemical analysis of signaling elements key to Wnt3a regulation of the canonical pathway. Based upon these biochemical determinations, we speculate that Wnt3a action does not catalyze dramatic changes in the composition of the signaling complexes that include these key signaling molecules. Rather, Wnt3a acts to traffic these relatively stable complexes from one cellular local to another, as evidenced by changes in the content of these molecules in well-defined subcellular fractions prepared from Wnt3a-responsive cells (F9 and HEK 293 cells alike). With the detailed characterization of these effects of Wnt3a on trafficking of Axin, Dvls, GSK3β and β-catenin in mammalian cells in cultures, the biochemical basis for how Wnt3a regulates the trafficking of these molecules during the activation of the canonical pathway becomes addressable.

## Methods

### Materials

The following reagents were purchased from the indicated commercial supplier(s): anti-Dvl1, Dvl2 and Dvl3 antibodies from Santa Cruz Biotechnology (Santa Cruz, CA); anti-GSK3β antibody from Cell Signaling (Danvers, MA); anti-Axin, anti-protein phosphatase 2A (PP2A) C subunit, and anti-phosphoserine-9-GSK3β antibodies from Upstate Biotechnology (Lake Placid, NY); anti-β-catenin antibody from Sigma (St. Louis, MO); anti-glyceraldehyde-3-phosphate dehydrogenase (GAPDH), anti-Na^+^-K^+^-ATPase, and anti-fibrillarin antibodies from Abcam (Cambridge, MA); anti-cytokeratin endoA antibody TROMA-1 from the University of Iowa Developmental Studies Hybridoma Bank (Iowa City, IA), Immobilon membrane from Millipore (Bedford, MA) ;and, purified, biologically active Wnt3a and anti-Axin antibody from R& D Systems (Minneapolis, MN). All BRET parent vectors including pGFP2-N1, N2, N3, C1, C2, or C3 and pRluc-N1, N2, N3, C1, C2, or C3 were purchased from BioSignal Packard (Montreal, Canada).

### Cell culture and transfection

The mouse F9 teratocarcinoma cells (F9) and human embryonic kidney 293 (HEK) cells were obtained from ATCC collection (Manassas, VA) and grown in Dulbecco's modified Eagle's medium (DMEM) supplemented with 15 % fetal bovine serum and penicillin/streptomycin at 37°C in 5 % CO_2_. Cells were transfected transiently with an expression vector (pCDNA3) harboring the rat Frizzled-1 (Rfz1) using lipofectamin (Invitrogen, Carisbad, CA) according to the commercial instructions. Cells were stimulated with purified Wnt3a at a final concentration of 20 ng/ml.

### Formation of primitive endoderm (PE)

Mouse F9 cells were transiently transfected to express Rfz1 (above) and then stimulated with purified Wnt3a for three days. Cells were cultured in DMEM medium for two more days and then were lysed in a RIPA buffer [50 mM Tris-HCl, pH 7.2, 150 mM NaCl, 5 mM EDTA, 2 mM Na_3_VO_4_, 1% NP-40, 0.25 % sodium deoxycholate and 0.05 % SDS, 1 mM phenymethysulfonyfluoride (PMSF), 10 μg/ml leupeptin, and 10 μg/ml aprotinin]. Cell extracts were analyzed for expression of the primitive endoderm-marker protein cytokeratin endoA by immunoblotting and staining with the anti-endoA, TROMA-1 monoclonal antibody.

### Lef/Tcf-sensitive transcriptional reporter gene assay

F9 cells were grown on 12-well plates and co-transfected with Rfz1 and either Super8xTOPFlash (M50) or the control plasmid harboring a β-catenin-insensitive promoter, Super8xFOPFlash (M51) [53]. After 1–2 days transfection, cells were treated with Wnt3a for up to 8 hr and then harvested and washed with phosphate-buffered saline (PBS) two times. Cells were harvested and lysed in a reporter gene lysis buffer [12.5 mM Tris-H_3_PO_4 _pH 7.8, 1 mM trans-1, 2-cyclohexanediaminetetraacetic acid (CDTA), 2 mM DTT, 10% glycerol and 1 % Triton X-100 (Promega, Madison, WI)]. Cell extracts were assayed for luciferase activity according to the manufacture's instructions (Stratagene, La Jolla, CA). Luciferase activity was normalized to the protein concentration of the cell lysates.

### Disruption & subcellular fractionation of cells

Cells propagated in P150 Petri dishes for optimal growth were either untreated or stimulated with purified Wnt3a for the times indicated in the figure legends. Cells were washed with PBS twice and harvested. Cells underwent disruption and subcellular fractionation as described previously [54]. Briefly, cells suspended in ice-cold buffer A (10 mM Hepes, pH 7.0, 5 mM MgCl_2_, 25 mM KCl, 1 mM Na_3_VO_4_, 1 mM PMSF, 10 μg/ml leupeptin, and 10 μg/ml aprotinin) were disrupted by repeated passage (10-times) through a 23-gauge needle and then mixed immediately with an equal volume of ice-cold buffer A containing of 0.25 M sucrose. Nuclei and unbroken cells were collected by centrifugation at 500 × *g *for 10 min. These cell pellets were resuspended again in an equal volume of ice-cold buffer A containing 0.1 % of NP-40 and then homogenized again by passage through a 23-gauge needle ten times. Nuclei were isolated by centrifugation at 500 × *g *for 10 min, washed once with buffer A containing of 0.1 % of NP-40, and lysed in a lysis buffer (10 mM Hepes, pH 7.0, 0.5 M KCl, 1.5 mM MgCl_2_, 0.2 mM EDTA, 1 mM DTT, 1 mM PMSF, 10 μg/ml leupeptin, and 10 μg/ml aprotinin). The subcellular fractions enriched in nuclei (NU) were prepared by centrifugation. After nuclei and unbroken cell were removed by centrifugation, EDTA was added to a final concentration of 10 mM and the mixture subjected to centrifugation at 16,000 × *g *for 15 min. The resultant pellets were washed once with buffer A containing of 0.25 M sucrose and lysed in a RIPA buffer (50 mM Tris-HCl, pH 7.2, 150 mM NaCl, 5 mM EDTA, 2 mM Na_3_VO_4_, 1% NP-40, 0.25 % sodium deoxycholate and 0.05 % SDS, 1 mM PMSF, 10 μg/ml leupeptin, and 10 μg/ml aprotinin). These fractions represent the standard subcellular fractions enriched in mitochondria + microsomes (M&M). The supernatant fractions then were centrifuged at 100,000 × *g *for 1 hr. The 100,000 × *g *supernatants represent the standard subcellular fraction enriched in cytosol (CY). The 100,000 × *g *pellets were washed with buffer A containing of 0.25 M sucrose once and lysed in a buffer (10 mM Tris-HCl, pH 7.5, 5 mM EDTA, 150 mM NaCl, 2 mM Na_3_VO_4_, 0.5 % NP-40 and 1 % Triton). This fraction represents the standard subcellular fraction enriched in plasma membrane (PM). A sample (5%) of the whole-cell suspension typically was lysed directly in RIPA buffer for determinations of total cellular protein content, of quantification of marker proteins, and of protein yields.

Protein content was determined by use of the Bradford assay. The purity of these enriched subcellular fractions was established by subjecting aliquots of each fraction to SDS-PAGE and the resolved proteins to immunoblotting. The immunoblots were stained with antibodies against each of the following protein markers employed routinely to establish the relative purity of subcellular fractions: anti-Na^+^-K^+^- ATPase (for plasma membrane), anti-GAPDH (for cytoplasm), and anti-fibrillarin (for nucleus).

### Immunoblotting

Proteins (30–100 μg protein/lane) were analyzed routinely on 8 % SDS-polyacrylamide gels (polymerized overnight) and the resolved proteins were transferred electrophoretically to Immobilon membrane "blots". The blots were stained with the antibodies indicated in the figure legends. Immune complexes were made visible using a horseradish peroxidase-conjugated, secondary antibody in tandem with ECL chemiluminescence. For Axin analysis, two distinct epitope antibodies were applied. Phosporylation (Ser-9) of GSK3β was used as physiological indicator of GSK3β.

### Quantification of stained proteins on immunoblots

Exposed films were scanned by calibrated Umax 1000 absorbance scanner equipped with SilverFastAi software (LaserSoft Imaging Inc. Longboat Key, FL). The bands were quantified by use of Aida software (Raytest, Germany).

### Analysis of protein-protein interactions by BRET

For routine BRET^2 ^measurements, cells were washed once with PBS, detached with 5 mM EDTA in PBS, and resuspended in BRET assay buffer (1× PBS buffer, plus 0.1% glucose) 48 h after transfection, as previously described [38]. The cells were treated with Wnt3a (20 ng/ml) for 15 min and readings of *Renilla *luciferase and GFP2 activity were then collected using a multidetector plate reader Mithras LB940 (Berthold Technologies, Oak Ridge, TN) after addition of assay substrate, Deep Blue coelenterazine (DeepBlueC, BioSignal Packard, Meriden, CT). Emission filters with 400 nm and 515 nm were used to detect *Renilla *luciferase (Rluc) and green fluorescence protein 2 (GFP2) activities, respectively. The BRET signal was determined by calculating the ratio of the light emitted by GFP2-fusion protein (515 nm) over the light emitted by Rluc-fusion protein (400 nm). The background signal was determined in cells in which an Rluc construct alone was expressed. The *XhoI *and *HindIII *cloning sites of pRluc-N3 were employed for creation of Rluc fusion proteins of interest. Dvl2-GFP2 constructs were prepared by cloning mouse Dvl2 cDNA into the *HindIII-BamHI *site of pGFP2-N2, respectively. GSK3-GFP2 was constructed by cloning human GSK3β cDNA into the *EcoRI-ApaI *site of pGFP-C3. All of above constructs were created with haemagglutin (HA) tags on their N- termini. β-catenin-Rluc and β-catenin-GFP2 were constructed by ligating the human β-catenin cDNA into the *KpnI-BamHI *site of pRluc-N1 and pGFP2-N1, respectively. Axin-Rluc was prepared by cloning the mouse Axin I cDNA into the *MluI-SacII *site of pRluc-N3 vector. The sequence of all constructs was confirmed by DNA sequencing.

## Abbreviations

APC : *Adenomatous polyposis coli*;

DMEM : Dulbecco's modified Eagle's medium;

Dsh/Dvl : Dishevelled;

GAPDH : Glyceraldehyde-3-phosphate dehydrogenase;

GSK3 : Glycogen synthase kinase-3;

Iso : Isoproterenol;

PBS : Phosphate-buffered saline;

PE : Primitive endoderm;         

Pro : Propranolol;

Rfz1: Rat Frizzled-1;

SDS-PAGE: Sodium dodecyl sulfate-polyacrylamide gel electrophoresis.

## Competing interests

The author(s) declare that they have no competing interests.

## Author's contributions

NY designed the study, analyzed the data and drafted the manuscript. DY carried out the BRET analysis and luciferase assay in HEK 293 cells. CCM participated in coordination and helped to draft the manuscript. All authors read and approved the final manuscript.
